# Plasticity varies with boldness in a weakly-electric fish

**DOI:** 10.1186/s12983-016-0154-0

**Published:** 2016-06-06

**Authors:** Kyriacos Kareklas, Gareth Arnott, Robert W. Elwood, Richard A. Holland

**Affiliations:** School of Biological Sciences, Queen’s University Belfast, Medical Biology Centre, 97 Lisburn Road, Belfast, BT9 7BL UK; School of Biological Sciences, Bangor University, Deiniol Road, Bangor, Gwynedd LL57 2UW UK

**Keywords:** Boldness, Behavioural plasticity, Individual variation, Weakly-electric fish

## Abstract

**Background:**

The expression of animal personality is indicated by patterns of consistency in individual behaviour. Often, the differences exhibited between individuals are consistent across situations. However, between some situations, this can be biased by variable levels of individual plasticity. The interaction between individual plasticity and animal personality can be illustrated by examining situation-sensitive personality traits such as boldness (i.e. risk-taking and exploration tendency). For the weakly electric fish *Gnathonemus petersii*, light condition is a major factor influencing behaviour. Adapted to navigate in low-light conditions, this species chooses to be more active in dark environments where risk from visual predators is lower. However, *G. petersii* also exhibit individual differences in their degree of behavioural change from light to dark. The present study, therefore, aims to examine if an increase of motivation to explore in the safety of the dark, not only affects mean levels of boldness, but also the variation between individuals, as a result of differences in individual plasticity.

**Results:**

Boldness was consistent between a novel-object and a novel-environment situation in bright light. However, no consistency in boldness was noted between a bright (risky) and a dark (safe) novel environment. Furthermore, there was a negative association between boldness and the degree of change across novel environments, with shier individuals exhibiting greater behavioural plasticity.

**Conclusions:**

This study highlights that individual plasticity can vary with personality. In addition, the effect of light suggests that variation in boldness is situation specific. Finally, there appears to be a trade-off between personality and individual plasticity with shy but plastic individuals minimizing costs when perceiving risk and bold but stable individuals consistently maximizing rewards, which can be maladaptive.

**Electronic supplementary material:**

The online version of this article (doi:10.1186/s12983-016-0154-0) contains supplementary material, which is available to authorized users.

## Background

Variation in behaviour between individuals has been shown extensively in many animal populations and linked to the way animals cope with their environment [1, 2]. Often, the variation is indicated on a continuum ranging from the lowest to the highest level of behavioural response within the population [3] and as such indicates the degree each individual exhibits the behaviour in relation to the rest of the population. This variation can be consistent across contexts (i.e. functional behavioural categories such as feeding), situations (i.e. sets of current conditions such as feeding with and without predators) and time [4–6]. Each behaviour that is consistently variable between individuals is termed an *animal personality trait* and a number of such traits can be used to describe personality in animals [7]. One of the most examined animal personality traits is *boldness*, which is indicated on a shy–bold axis [8]. Human-derived terminology defines boldness as the consistent willingness to take risks in unfamiliar situations [9]. This definition is often appropriated when studies consider its evolutionary and ecological consequences [10]. However, ‘ecologically-based’ approaches typically define bolder individuals as those that are the least affected by risk and more willing to approach and explore novel objects or environments [11, 12].

Boldness, like all personality traits, remains consistent depending on the degree in which behavioural plasticity varies between individuals [13]. On one hand, individuals can adjust their behaviour, but the extent of adjustment may be relatively uniform within the population. Thus, even if mean levels of behaviour change, between-individual variation is maintained, i.e. all individuals show similar plasticity [14]. For example, the mean boldness (propensity to exit shelter) of salamander larvae decreases in the presence of predators, but the variation between individuals is maintained across situations with and without predators [15]. On the other hand, environmental changes can affect the behaviour and physiology of some individuals more than others [16, 17], e.g. rainbow trout that exhibit lower activity and aggressiveness are affected more by increasing environmental stressors [18]. Consequently, behavioural variability within populations can be biased by the variable degree in which environmental changes affect individuals. Individuals may be more or less flexible over an environmental gradient of changing conditions, i.e. they exhibit variable levels of *individual plasticity* [19].

Links between personality and individual plasticity have been reported when testing boldness across situations varying in their level of risk and familiarity [20]. Lima and Bednekoff suggest that behavioural response depends on the level of perceived risk, which can vary between individuals [21]. A greater response can thus be associated with a greater perception of risk, even when uncertain about its presence, while the ability to adjust response, depending on risk levels, can be overall more beneficial for surviving in the wild [22]. This manifests in risk-taking behaviour, with individuals that respond more to risk (i.e. those taking less risk) also showing greater changes across shifting levels of perceived risk. For example, between situations that vary in perceived predatory risk (presence or absence of sparrowhawk model), shy chaffinches (least active in a novel environment) show greater behavioural plasticity than bold chaffinches (most active in a novel environment) [23]. Mortality, growth and fecundity can all be affected by an individual’s response to changes in risk [24], e.g. shier damselfish show lower mortality rates by being less active in unfamiliar environments [25]. It is therefore imperative to examine how changes in levels of perceived risk can affect boldness and individual plasticity.

For weakly-electric fish, the level of perceived risk in their environment is most significantly affected by light conditions. Most species prefer lower light transmission, where they can integrate their electric-sensing with other senses in the absence of light [26, 27]. One example is the Central African mormyrid *Gnathonemus petersii*, which favours nocturnal activity and turbid, vegetated waters [28, 29]. This species can perceive spatial features, navigate and explore objects and environments by using active electrolocation, i.e. the sensing of changes to a self-produced electric discharge [30, 31]. Though often being prey to bigger electric fish, it is argued that a function of electrolocation is avoiding risk from visually-guided predators in darker environments [31, 32]. The lower predation risk would increase their motivation to approach and explore objects and environments, hence their preference to be active in the dark [26, 27]. However, the change in motivation can be greater in some individuals, depending on how plastic they are, which can affect mean boldness levels. This is supported by evidence of differences between individuals in the degree of change in food searching times across light conditions [32]. The aim of the present study was to examine boldness and changes in boldness across situations, with a particular interest in the effect of light conditions on individuals.

Boldness was indicated by the willingness of *G. petersii* to approach (latency times) and inspect (exploration times) novel objects and environments. First, fish were tested with a different novel object on four occasions, to control for differences in object characteristics. The tests were carried out in a bright, familiar environment. Then, individuals were tested in two separate novel-environment situations differing in light condition, i.e. a dark and a bright novel-environment. Finally, an intra-individual variance statistic was used to measure individual plasticity across the environmental gradient between bright and dark [19, 33]. It was tested whether boldness from the novel-object tests **1)** was consistent with boldness in the bright and dark novel-environment situations and **2)** related to individual plasticity across these novel-environment situations.

## Methods

### Animal maintenance and housing

Twelve juvenile (70–100 mm length), wild-caught *G. petersii* of unknown gender (external sexual dimorphism is lost in captivity) [34] were imported and commercially supplied by Grosvenor’s Tropicals, Lisburn, Northern Ireland. Fish were housed individually in ~25 L of water, fed 15–20 chironomid larvae daily and kept on a 12 h:12 h light to dark photoperiod. Housing tanks were enriched with shelter (plastic pipes), sediment and plastic plants, stones and ceramics. Housing and experimental tanks were fitted with filtering and heating equipment and kept on same-level benches. Water quality in all tanks was tested twice-weekly and maintained by partial water changes (mixed tap and reverse osmosis water). The pH was kept at 7.2 ± 0.4, temperature at 26 ± 1^o^ and conductivity at a range between 150–300 μS/cm.

### Behavioural tests

#### Test conditions and procedures

Light conditions varied between those within (*bright light* at 350–600 nm and 300 lux at water surface) and those exceeding (*dark* in infra-red light >800 nm and 0 lux at water surface) the visible spectrum of *G. petersii* [35]. Water conductivity in the test tanks was 150 ± 50 μS/cm. External cues were limited by attaching visual barriers (opaque blue plastic sheets) around both the novel-environment test tanks and the housing tanks, during testing. Behavioural variables were measured live during the novel-object test and from recordings of the novel-environment test. This was carried out by a single observer (KK), with a response latency of 1–2 s, using a stopwatch with a ±0.2 s measuring error.

#### Novel-object tests

Novel-object tests were in bright light. These were carried out following a 2 week acclimatisation period to ensure that the objects were novel to the fish, but not the environment (housing tank). Each individual received four separate novel-object tests, with a 5 min interval between each test. The test was repeated with different novel objects in order to control for variation in potential effects elicited by the differences in the characteristics of novel objects. These effects could result from how each object is perceived by individuals. *G. petersii* can sense multiple properties of objects, some of which are typically not perceived by non-electrosensing fish, such as resistance and capacitance [29]. To that end, the novel objects not only differed in shape, colour and size, but also material. Objects included: a ~ 5 cm long black fishing weight (A), a ~7 cm long stainless-steel fishing lure without the hook (B), a ~15 cm long yellow/green plastic dinosaur toy (C) and a 10 cm^3^ multicolour wooden cubic toy attached to a small brass weight (D). Following recommendations from Wilson et al. [36], objects were presented to each fish in the same order (A-B-C-D) to control for carryover effects. The objects were lowered in housing tanks at the furthest non enriched area from the individual’s shelter using a monofilament-line pulley-system. Fish were given up to five minutes to approach each object (within ~1.5 body-lengths), which was measured as latency time [11]. Then a further 1 min was allowed for exploration (75 % of individuals explored new objects under 55 s in preliminary studies; see Additional file 1), during which the time spent performing electrosensing movements (motor probing acts, e.g. lateral and chin probing) [37] within the 1.5 body-length distance was measured as exploration time.

#### Novel environment tests

The recording of the novel-environment tests was carried out both under bright light and in the dark and started a week after the novel-object tests (overall 3 weeks in the laboratory), which allowed individuals to acclimatise to laboratory light conditions. Timers switched between bright light and dark photoperiods every 12 h (lights went on at 7 am and off at 7 pm), daily. Novel-environment tests were carried out with a random light-condition order between fish. Individuals randomly selected to be tested first in the dark, were tested between 5 am and 6 am and then in bright light between 8 am and 9 am. Those randomly selected for being tested first in bright light, were tested between 5 pm and 6 pm and then in the dark between 8 pm and 9 pm. This procedure of recording during normal laboratory photoperiods controlled for the risk of effects from circadian rhythms [31]. Each individual was introduced to a segregated housing section (30 cm Length by 30 cm Width and 30 cm Height, ~27 L) of the experimental tank with shelter and enrichments. Here, individuals were allowed to habituate for ~12 h prior to their first novel-environment test, and ~2 h during photoperiod changes between tests (~ an hour before and ~ an hour after lights turned on or off). Tests began by lifting the plastic opaque divider creating the housing section via a pulley system, allowing the fish entry to the rest of the tank (60 cm Length by 30 cm Width and 30 cm Height, ~54 L). This area constituted the novel environment and included items that were similar to enrichments in their housing tanks i.e. shelters (plastic pipes), ceramics, stones and plastic plants of variable sizes. The items within the novel area were rearranged and/or replaced between bright and dark tests for all fish. A wall-mounted infra-red camera provided a live feed of the entire novel-environment test-tank from a birds-eye view. This was relayed through a recorder to a computer placed out of view from the tank. During recording, fish where allowed up to a maximum of 1 h to enter the novel environment (i.e. until an individual’s tail passed the mark on the bottom of the tank) and a further 10 min to explore. During the later viewing of the recordings, latency time was measured until an individual entered the novel environment or until the hour-mark was reached, in which case latency was recorded at 3600 s and exploration at 0 s (this was the case for only one individual in the bright novel environment). Exploration was measured as the time actively moving in the novel area and performing electrosensory probing acts.

### Analysis

Calculations, statistical analyses and graphical representations were all produced in Minitab® statistical software (version 17; Minitab Inc., State College, PA). Data from the novel-object tests were either normally or approximately normally distributed. Only exploration times from the novel-environment test data were normally distributed. Measures were summed to produce composite, standardized boldness scores. This was carried out by adding positive (time exploring) and subtracting negative (latency time to approach) indicators and then standardising (*z*-scores).

**Fig. 1:**
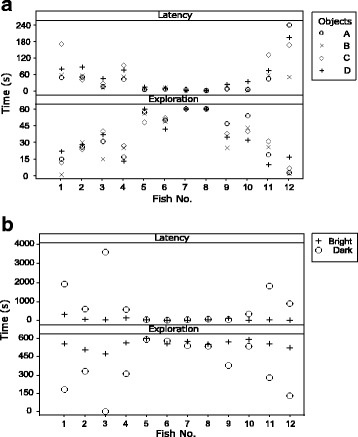
Latency and exploration times for each individual, as measured in all novel-object tests (**a**) and each of the novel-environment situations (**b**). Individuals that were more explorative, were also less latent to approach objects. Similarly, some individuals were more explorative and less latent in the bright novel environment. However, in the dark novel environment individuals were overall more explorative and less latent

In novel-object tests, some individuals were both less latent to approach and more explorative than others (Fig. 1a). Preliminary analyses on the novel-object tests indicated a strong linear relationship between latency and exploration (*R*^*2*^ = 0.500, *F*_*1,47*_ = 47.32, *P* < 0.01). Even though some differences were apparent between objects (Fig. 1a), these were not significant (*R*^*2*^ = 0.065, *F*_*3,47*_ = 2.04, *P* = 0.122). This suggested that boldness levels were indicated by both measures with no effect from object characteristics. Measures from all four novel-object tests were, thus, used to create boldness scores. Inter-individual differences in latency and exploration were not similar between bright and dark novel environments (Fig. 1b). Separate boldness scores were produced for each novel-environment situation, dark and bright. Composite scores were used to test consistency in boldness across novel-environment situations and between novel-environment and novel-object situations. For this, two Linear Regression models (LR) were used. The first (LR1) tested the relationship between bright and dark novel-environment scores. The second (LR2) tested if the effect of situation also affected how novel-environment scores related to novel-object scores, i.e. were predicted by situation, dark or bright, and its interaction with novel-object scores.

To calculate individual plasticity statistics, typically a measure of each individual’s variance between two situations is used [38]. Following Asendorpf’s [33] suggestions, here, this was measured as the intra-individual variance (*Var*) of each fish such that

$$ Va{r}_{xy}=\frac{{\left({z}_x-{z}_y\right)}^2}{2} $$

where *z* is the standardized phenotypic score (here the novel-environment boldness score) at situation *x* (bright) and *y* (dark). Higher intra-individual variance values designated greater degree of change and therefore greater individual plasticity. In order to test if individual plasticity varied with boldness, intra-individual variance statistics were then correlated with novel-object boldness scores (Spearman’s, *r*_*s*_).

## Results

**Fig. 2:**
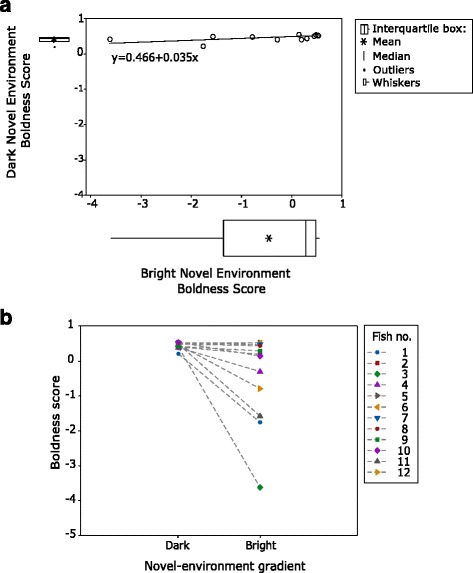
Comparisons between the bright and dark novel environment. The marginal plot (**a**) shows an average increase in boldness and a decrease in variability in the dark novel environment (box-plots), but also no significant linear relationship between boldness scores from the two novel-environment situations (regression). The individual line plot (**b**) shows some individuals changing more than others between bright and dark

**Fig. 3:**
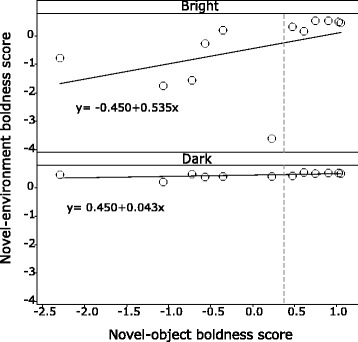
Linear relationships in boldness between the novel-object situation and each of the novel-environment situations, bright and dark. Novel-object boldness scores were significantly more consistent with those in the bright than those in the dark environment. Those with novel-object boldness scores below the median (dotted line) showed more change between light and dark

**Fig. 4:**
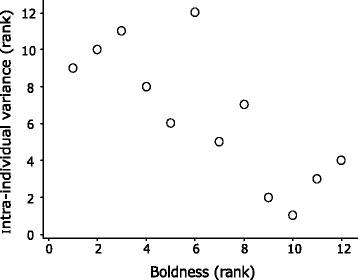
Rank correlation between intra-individual variance and boldness scores from the novel-object tests. Bolder individuals were less plastic between the bright and dark novel environment

Individual scores were not consistent between novel-environment situations (LR1, *R*^*2*^ = 0.251, *F*_*1,11*_ = 3.35, *P* = 0.097) (Fig. 2a). Boldness was significantly different between the bright and dark novel environment (LR2, *R*^*2*^ = 0.211, *F*_*1,23*_ = 6.85, *P* = 0.016), being on average greater and less variable in the dark (*x̄*=0.45, *s* = 0.09) than in the bright (*x̄*= -0.45, *s* = 1.28) novel environment (Fig. 2a). However, the change between bright and dark was greater for some fish (Fig. 2b). Those with the greater change were also ones with below-median novel-object boldness (Fig. 3). The change between bright and dark affected the relationship between novel-object and novel-environment scores (LR2, interaction: *R*^*2*^ = 0.143, *F*_*1,11*_ = 4.65, *P* = 0.043), which was stronger with the bright than the dark novel-environment scores (Fig. 3). The intra-individual variance in boldness between the two novel-environment situations was strongly negatively correlated with boldness score from the novel-object tests (Spearman’s, *r*_*s*_ = -0.776, *P* = 0.003) (Fig. 4).

## Discussion

This study provides compelling evidence supporting the hypothesis that the degree of individual plasticity varies significantly with personality. Boldness was inconsistent between bright and dark novel-environments (Fig. 2a) and the intra-individual variance exhibited across these environments depended on boldness (Fig. 4). However, when maintaining bright light conditions, changes in levels of familiarity/novelty (whether it is a single unfamiliar object in a familiar environment or a completely unfamiliar environment) seem to have little effect on behavioral variability between individuals (Fig. 3a). These findings emphasize the overwhelming effect of light condition and indicate a boldness trait which is specific to higher risk situations, given that bright light is naturally avoided by *G. petersii* [27].

An indirect effect of the environment can be seen when regularly changing conditions (e.g. light, temperature and turbidity) influence the motivational state of individuals. For example, small within-day increases in temperature relate to an increase in the tendency of damselfish to exit a shelter (measure of boldness), but more so in some individuals than others [39]. It is suggested that an increased motivation to exit shelter and look for food can be associated with the need to compensate for the increased metabolic rates under elevated temperatures [39, 40]. The present study reaffirms that a similar effect is induced by perceived risk through manipulations of light. The decrease in risk in the dark (lower predator threat) increases the motivation to explore a novel environment in some individuals and as a result impacts mean boldness in that situation. Notably, the results presented here also show that the effect varies with boldness (Fig. 3), i.e. perceived risk affects the motivation of shier individuals more. Motivation levels can vary as a function of personality [41] and therefore the impact on motivation by changing conditions may also vary depending on personality traits like boldness.

The negative relation between boldness and individual plasticity (Fig. 4) indicates trade-offs that enable bolder individuals to out-compete shier ones (e.g. for food) in higher-risk situations. However, maintaining bold behaviour in risky situations can be disadvantageous and in the long-term maladaptive [42]. Shier individuals, which are more responsive to change and more plastic [43], gain less when risks are high but compensate in safer environments. This manifests in the behaviour of *G. petersii*, which is more variable in situations with greater selective pressure (i.e. in bright light with high predatory risk) where risk-aversion is elicited in shier fish, while in the safe dark situation boldness scores are overall high (Fig. 2).

The selection of plastic or consistent behaviour with changing conditions can depend on both the physiological and cognitive state of individuals [44, 45]. Differences between individuals in their physiological stress response [16, 17] and cognitive risk-assessment [22] can explain the differences in strategy, i.e. plastic boldness vs. stable boldness [46]. For example, recent evidence suggests that bolder fish make faster decisions [47]. There is therefore a need to examine mechanisms further, including those used for sensing and processing information, and test how they relate to individual plasticity and personality.

## Conclusions

The current study highlights that individuals can vary in the degree of behavioural plasticity exhibited between situations differing in risk level depending on their position along an important animal personality axis, the shy-bold continuum. This strongly suggests that the ability to cope with changing conditions, especially ones associated with the perception of risk, vary between individuals as a function of their personality. Finally, it accentuates that individual variation can be a significant predictor of behaviour and behavioural change in wild populations.

## Additional file

Additional file 1: Datasets and calculated statistics. The file includes: 1) datasets of recordings from preliminary and experimental (novel object and novel environment) tests, and 2) tables with calculated boldness scores and intra-individual variance statistics. (XLSX 15 kb)
